# SCC*mec* Genotypes of Methicillin-Resistant *Staphylococcus aureus* in Nasal Carriage of Multiple Sclerosis Patients in Iran

**Published:** 2019-12

**Authors:** Yasaman JAMSHIDI, Mohammad Reza POURMAND, Zahra PAKBAZ, Amirhossein POURMAND, Abbas RAHIMI FOROUSHANI, Mohammad Ali SAHRAIAN

**Affiliations:** 1. Department of Pathobiology, School of Public Health, Tehran University of Medical Sciences, Tehran, Iran; 2. Urology Research Center, Tehran University of Medical Sciences, Tehran, Iran; 3. Department of Epidemiology and Biostatistics, School of Public Health, Tehran University of Medical Sciences, Tehran, Iran; 4. MS Research Center, Neuroscience Institute, Department of Neurology, Sina Hospital, Tehran University of Medical Sciences, Tehran, Iran

**Keywords:** Multiple sclerosis, Methicillin-resistant *Staphylococcus aureus*, SCC*mec* typing

## Abstract

**Background:**

Asymptomatic nasal colonization of Methicillin-Resistant *Staphylococcus aureus* is common in Multiple Sclerosis patients. SCC*mec* types I to III are mainly attributed to HA-MRSA strains whereas SCC*mec* types IV and V have commonly been reported in CA-MRSA infections. Here, we assessed the frequency of nasal carriage of MRSA in MS patients. This study aimed to evaluate MRSA SCC*mec* typing in MS nasal carriage.

**Methods:**

A cross-sectional descriptive study was conducted from Feb and Jun 2017 in MS Research Center, Tehran University of Medical Sciences (TUMS), Iran. Overall, 620 nasal swabs were collected (325 from MS patients and 295 from control group). Antimicrobial susceptibility test was performed using the disk diffusion and E-test method. Presence of *mecA* gene was confirmed by PCR assay and multiplex PCR was performed for SCC*mec* typing of MRSA isolates.

**Results:**

The frequency of MRSA among the MS patients and control group was almost equal (9.2% and 10.1%, respectively). SCC*mec* typing detected only types III, IV and V in both groups and type IV was the most predominant type in MS patients and control group. SCC*mec* type III was more prevalent in control group than MS patients (40% vs. 20%). Moreover, the frequency of SCC*mec* type V in MS patients was significantly higher than control group (36.7% vs. 3.3%).

**Conclusion:**

Although most MRSA isolates were collected from inpatients, interestingly there is a high frequency of SCC*mec* types IV and V in MS group. Moreover, MRSA isolates were not resistant to more antibiotics in *SCCmec* type III than types IV–V.

## Introduction

Multiple Sclerosis (MS) is an inflammatory demyelinating autoimmune disease that involves the central nervous system (CNS) and usually affects people between the ages of 20 and 50 yr (1). The etiology of MS is currently unknown. Genetic predisposition, T cell activation and environmental factors such as various infections have been contributed to the initiation and exacerbation of this autoimmune disease (2). Infectious diseases appear to play an important role in activating peripheral autoreactive T cells. Accordingly, development of MS is frequently associated with acquisition of bacterial and viral infections (3, 4).

Infections caused by Staphylococcus aureus are of serious public health concern throughout the world. *S. aureus* is one of the major human pathogens, responsible for a wide range of infections from mild to life-threatening conditions (5)**.** This pathogen is increasingly showing resistance to multiple antimicrobial agents (6). Methicillin-resistant Staphylococcus aureus (MRSA) is an opportunistic pathogen characterized by the development in virulence and antibiotic resistance (7, 8).

MRSA can colonize multiple body sites of human host, but the anterior nares are frequently being colonized in approximately 30%–50% of individuals, moreover, the host can be predisposed to a wide range of infections by asymptomatic carriage of MRSA (9). Nasal colonization of MRSA can be a risk factor for subsequent infection when the balance of host-pathogen is disrupted (10). Although MRSA has been reported as a main hospital-acquired pathogen (HA-MRSA) worldwide, in the last two decades community-acquired methicillin-resistant *S. aureus* (CA-MRSA) infections has emerged (11). SCC*mec* types I to III mainly attributed to HA-MRSA strains are associated with multidrug resistance (MDR). SCC*mec* types IV and V have commonly been reported in CA-MRSA infections (12, 13).

MRSA originates from methicillin-susceptible *S. aureus* by acquisition of staphylococcal cassette chromosome *mec* (SCC*mec*) element. The SCC*mec* consists of two essential loci, the *ccr* gene complex (*ccr*) and the *mec* gene complex (*mec*) harboring the *mecA* gene. Penicillin-binding protein 2a, encoded by the *mecA* gene, can cause resistance to methicillin in staphylococci by reduction in affinity (14, 15). Molecular typing methods are essential for continuous surveillance and infection control programs, so it may help to prevent the dissemination of MRSA. Among various techniques used for genotyping of MRSA, SCC*mec* typing is considered as a simple and cost-effective method that can distinguish between the HA-MRSA and CA-MRSA strains (16, 17).

Here, we assessed the frequency of nasal carriage of MRSA in MS patients. The aim of this study was to evaluate MRSA SCCmec typing in MS nasal carriage.

## Materials and Methods

### Study population

A cross-sectional descriptive study was conducted from Feb and Jun 2017 in MS Research Center, Tehran University of Medical Sciences (TUMS), Iran. Overall, 325 MS patients and 295 apparently healthy individuals, as a control group, were included in the study.

Ethical approval for this study was obtained from TUMS ethics committee (Approval number: IR.TUMS.SPH.REC.1396.3054).

Samples were collected from persons without MRSA infection. A self-administered questionnaire was provided to each participant to collect demographic information, medical history and factors that potentially may be related to *S. aureus* nasal colonization and transmission.

### Sample processing and identification of bacterial isolates

Samples were collected from the anterior nasal cavities of each patient and healthy person for the isolation of *S. aureus* using cotton swab. Clinical isolates were cultured on the blood agar and mannitol salt agar and were identified by conventional biochemical tests including catalase, tube coagulase, mannitol fermentation and DNase enzyme production tests. *S. aureus* isolates were stored in freezer at −70 °C in tryptic soy broth (TSB) containing 20% glycerol.

### Antimicrobial susceptibility test

Antimicrobial susceptibility tests were performed using standard disc diffusion method according to guidelines recommended by the Clinical and Laboratory Standards Institute (CLSI, 2016). The following antibiotics were tested: cefoxitin (30 μg), clindamycin (2 μg), erythromycin (15 μg), ciprofloxacin (5 μg), doxycycline (30 μg), teicoplanin (30 μg), rifampin (5 μg), co-trimoxazole (1.25/23.75 μg) and mupirocin (200 μg) [MAST Diagnostics, Merseyside, UK]. The minimum inhibitory concentration (MIC) for vancomycin was determined by E-test (BioMerieux, Marcy l'Etoile, France) method.

### DNA extraction and identification of MRSA isolates

Genomic DNA was extracted from MRSA isolates by Genomic DNA Extraction Kit (YTA, Tehran, Iran) according to the manufacturer instruction. Isolates were confirmed as MRSA by cefoxitin disc screening test and PCR test for *mecA* gene.

### Multiplex PCR for SCCmec Typing

The standard SCC*mec* types strains were provided from Sina Molecular Laboratory, Tehran, Iran. Multiplex-PCR assay was performed for the typing of MRSA isolates. Different SCC*mec* types determined by 9 pairs of primers including the unique and specific primers for *ccr* gene complex and *mec* gene complex in order to determine types of SCC*mec* elements and a pair of primers for the *mecA* gene (18).

Multiplex PCR-1 was optimized by initial denaturation step (94 °C, 2 min), 35 cycles of denaturation (94 °C, 2 min), annealing (57 °C, 1.5 min), extension (72 °C, 2 min), and a final elongation at 72 °C for 2 min and holding at 4 °C. The PCR conditions of Multiplex PCR-2 were as follows: Initial denaturation (94 °C, 1 min), 30 cycles of denaturation (94 °C, 1 min), annealing (60 °C, 1 min), and extension (72 °C, 2 min) and holding at 4 °C. For Multiplex PCR-2, the reaction mixtures are the same as for Multiplex PCR-1 other than the concentration of MgCl2 and the primer pairs. The products were electrophoresed on 1% agarose.

### Statistical analysis

Statistical analysis was performed by using Chi-square and Fisher's exact test (SPSS software, version 24, Chicago, IL, USA). A *P-*value of <0.05 was considered statistically significant. Due to their similar biological features, we considered SCCmec III to be one group and SCCmec IV and SCCmec V to be another group.

## Results

### Study population

Overall, 620 nasal swabs were collected (325 from MS patients and 295 from the control group). The patient group comprised of 97 (29.85%) males and 228 (70.15%) females. The control group consisted of 104 (35.25%) males and 191 (64.75%) females. The mean age of MS patients and control group was 34.8 ± 11.2 and 30.5 ± 7.7 yr, respectively. Nasal carriage rate of *S. aureus* among MS patients was 36.3% (n= 118) while in control group it was 32.5% (n=96). There was no statistically significant association between *S. aureus* colonization and MS disease (*P* =0.7).

### Sample processing and identification of bacterial isolates

The frequency of MRSA among the MS patients and control group accounted for 30 (9.2%) and 30 (10.1%) individuals, respectively. Resistance to cefoxitin was detected in 60 isolates (9.7%). *mecA* gene amplification was observed in all MRSA isolates. MS patients and control group were not significantly different in the frequency of MRSA colonization (*P*=0.345). All the MRSA isolates from the study group were susceptible to teicoplanin and vancomycin but relatively resistant to clindamycin and erythromycin. In MS patients group, most isolates were susceptible to rifampicin (90%) and mupirocin (93.4%). In control group, the proportion susceptibility to mupirocin and cotrimoxazole was 90%.

### Multiplex PCR for SCCmec Typing

Multiplex PCR results for SCC*mec* typing revealed that the most prevalent SCC*mec* type in MS patients was type IV (43.3%, n=13), followed by type V (36.7%, n=11) and type III (20%, n=6). However, in healthy individuals group the rates of SCC-*mec* types IV, III and V were 56.7% (n=17), 40% (n=12) and 3.3% (n=1), respectively (Table 1). SCC*mec* type I and II were not detected in both groups.

**Table 1:** Association of SCCmec types with demographic features and antibiotic resistance patterns of MRSA isolate in MS patients and healthy individuals

***Variables***	***MS patients N(%)***			***Healthy individuals N(%)***							
	***Type III N= 6 (20%)***	***Type IV–V N= 24 (80%)***	***Type III VS IV–V P-value***	***Type III N= 12 (40%)***	***Type IV–V N= 18 (60%)***		***Type III VS IV–V P-value***
Mean Age (yr)		31.5 ± 2.6	36.6 ± 3.1	1.000	32.2 ± 1.7	29.7 ± 1.07	0.400
Sex	Female	3 (50%)	16 (66.7%)	0.641	9 (75%)	8 (44.4%)	0.098
	Male	3 (50%)	8 (33.3%)		3 (25%)	10 (55.6%)	
Inpatients^*^		6 (100%)	22 (91.7%)	1.000	4 (33%)	5 (27.8%)	1.000
Antibiotic usage		1 (16.7%)	12 (50%)	0.196	3 (25%)	0 (0%)	0.054
Nasal and Upper respiratory failure		0 (0%)	1 (4.2%)	1.000	2 (16%)	4 (22.2%)	1.000
Mean Disease Course (year)		5.6 ± 1.5	9.4 ± 1.9	1.000	-	-	-
Resistance to Erythromycin		6 (100%)	14 (58.3%)	0.074	11 (91.7%)	6 (33.3%)	0.005
Resistance to Clindamycin		6 (100%)	10 (41.7%)	0.019	10 (83.3%)	9 (50%)	0.121
Resistance to Doxycyclin		4 (66.7%)	10 (41.7%)	0.047	5 (41.7%)	3 (16.7%)	0.007
Resistance to Co-trimoxazol		2 (33.3%)	10 (41.7%)	1.000	1 (8.3%)	2 (11.1%)	1.000
Resistance to Ciprofloxacin		3 (50%)	8 (33.3%)	0.641	10 (83.3%)	1 (5.6%)	0.000
Resistance to Rifampin		3 (50%)	0 (0%)	0.005	9 (75%)	1 (5.6%)	0.000
Resistance to Mupirocin		1 (16.7%)	1 (4.2%)	0.366	1 (8.3%)	2 (11.1%)	1.000
Resistance to Vancomycin		0 (0%)	0 (0%)	-	0 (0%)	0 (0%)	-
Resistance to Teicoplanin		0 (0%)	0 (0%)	-	0 (0%)	0 (0%)	-

### SCCmec Type III vs. Types IV-V in MS Patients Group

In MS patients group, six MRSA isolates with SCC*mec* type III compared to 24 MRSA isolates with *SCCmec* type IV–V. SCC*mec* type III vs. types IV–V group had more males (33.3% vs. 50%), more inpatients (100% vs. 91.7%), but less average age (31.5 vs. 36.6 yr), less mean MS disease course (5.6 vs. 9.4 yr) and fewer individuals with history of antibiotic usage.

None of these differences were statistically significant. Antibiotics susceptibility patterns were different in two SCC*mec* types groups. There was a significant association between SCC*mec* types and susceptibility to rifampin, doxycyclin and clindamycin (Table 1).

### *SCCmec* Type III vs. Types IV–V in Control Group

However, there was no statistically significant association between *SCCmec* types and mentioned characteristics. In *SCCmec* type III group, resistance to some of antibiotics e.g. erythromycin, doxycycline, ciprofloxacin and rifampin were significantly higher than *SCCmec* types IV–V group.

## Discussion

The *S. aureus* nasal carriage in immunocompromised patients may have serious consequences because nasal colonization can cause an infection when the host-pathogen balance is disturbed.

MS patients have impaired T lymphocyte function, then, they are considered as immunocompromised patients. The *S. aureus* nasal carriage in immunocompromised patients may have serious consequences because nasal colonization can cause an infection when the host-pathogen balance is disturbed (19). The frequency of nasal carriage of *S. aureus* in MS patients was significantly higher than in healthy persons in other studies (20, 21). In present study, nasal carriage rate of *S. aureus* among MS patients (36.3%) was relatively similar to that of control group (32.5%). The resistance to antibiotics, especially to methicillin, in *S. aureus* has emerged as a major public health problem. Limited studies were conducted in nasal MRSA carriage in MS patients. The rate of MRSA in Iran was high and ranged from 20.4% to 90% in different areas of the country (22). In current study, the frequency of MRSA was 9.2 % and 10.1% among the MS patients and the control group respectively.

Higher rates, 22.2% were reported (20). The frequency of MRSA in MS patients was almost similar to another (9.3%) study (21).

The overall rates of *SCCmec* type were 50%, 30% and 20% for types IV, III and V, respectively. In MS patients, *SCCmec* type IV (43.3%) was the predominant, followed by type V (36.7%) and type III (20%). In control group, the frequency of types IV, III and V was 56.7%, 40%, 3.3%, respectively. We found that the frequency of type V is significantly higher in MS patients than control group (*P*=0.001). Although 93.3% (28/30) of MRSA isolates recovered from MS patients were collected from hospital, but surprisingly the molecular typing showed high prevalence of MRSA strains carrying SCC**mec** types IV and V (91.7%), traditionally attributed to CA-MRSA strains. SCC**mec** types I to III have been reported to be the most frequent nosocomial MRSA strains in the United States (23, 24), Europe (25, 26), and Switzerland (27, 28). In southern Iran, type III was the most prevalent (74.3), followed by type IV (12.1%), type V (2.6%) and type I (0.6%) (29). In another study in Iran, SCC**mec** types III (45%) and IVc (24%) were the most prevalent among HA-MRSA isolates (30).

In this study, we considered SCCmec III to be one group and SCCmec IV and SCCmec V to be another group (due to their similar biological features) and compared the demographic characteristics and antibiotics susceptibility pattern between two groups, in MS patient and control group, separately. Like other studies from Iran, most isolates were sensitive to vancomycin (100%) and teicoplanin (100%). The highest rate of resistance was observed for clindamycin and erythromycin. In MS patients group, resistance to clindamycin, doxycycline and rifampin was significantly higher in SCC*mec* type III vs. types IV–V group. In control group, resistance to erythromycin, doxycycline, ciprofloxacin and rifampin group was significantly higher in type III than in types IV–V group. Overall, the antimicrobial resistance patterns in MS patients and control group were relatively similar. In Iran, decreased sensitivity of HA-MRSA to erythromycin, clindamycin, co-trimoxasol, ciprofloxacin, cephalexin, tetracyclin, and gentamicin, predorminatly were reported among isolates harboring type III *SCCmec* gene (30). In Iran, all HA-MRSA isolates were susceptible to quinupristin–dalfopristin, linezolid, and vancomycin, but most isolates were resistant to penicillin (100%), erythromycin (50%), clindamycin (27%), and gentamicin (18%) (29). Vancomycin, quinupristin–dalfopristin, line-zolid and teicoplanin can still be used to treat different MRSA infections in Iran. In MS patients and control groups there is no statistically significant association between *SCCmec* types groups and demographic characteristics include, hospitalized patients, average age, history of antibiotic usage**,** nasal and upper respiratory failure.

## Conclusion

Although most MRSA isolates were collected from inpatients, interestingly there is a high frequency of SCC*mec* types IV and V in MS group. Moreover, MRSA isolates were not resistant to more antibiotics in *SCCmec* type III than types IV–V.

## Ethical considerations

Ethical issues (Including plagiarism, informed consent, misconduct, data fabrication and/or falsification, double publication and/or submission, redundancy, etc.) have been completely observed by the authors.
